# Prediction of Drill Bit Breakage Using an Infrared Sensor

**DOI:** 10.3390/s21082808

**Published:** 2021-04-16

**Authors:** Min-Jae Jeong, Sang-Woo Lee, Woong-Ki Jang, Hyung-Jin Kim, Young-Ho Seo, Byeong-Hee Kim

**Affiliations:** 1 Dry Strip Division Dry Strip System Group, PSK Inc., Hwaseong 18449, Korea; mj1925@psk-inc.com (M.-J.J.); hj2004@psk-inc.com (H.-J.K.); 2 Interdisciplinary Program in Biohealth-Machinery Convergence Engineering, Kangwon National University, Chuncheon 24341, Korea; lswoo@kangwon.ac.kr (S.-W.L.); mems@kangwon.ac.kr (Y.-H.S.); 3 The Advanced Education Center of Convergence Technology in Personalized Smart Healthcare System for Active Seniors, Kangwon National University, Chuncheon 24341, Korea; wkddndrl@kangwon.ac.kr

**Keywords:** tool condition monitoring, drill tool wear, drill tool breakage, infrared sensor

## Abstract

In this paper, a novel drill bit breakage prediction method featuring a low-cost commercial infrared sensor to monitor drill bit corner wear is proposed. In the proposed method, the drill bit outer corner wear state can be monitored by measuring reflected infrared light because the reflection phenomenon is influenced by wear, edge shape, and surface roughness of the drill bit. In the experiments, a titanium workpiece was drilled without using cutting fluid to accelerate drill bit fracture. After drilling a hole in the workpiece, reflected infrared light was measured for the drill bit rotating at 100 rpm. Collected data on intensity of infrared light reflected from the circumferential surface of the drill bit versus the rotation angle of the drill bit were considered to predict tool breakage; two significant positions to predict tool breakage were found from the reflected infrared light graphs. By defining gradient vectors from the slopes of the reflected infrared light curves, a reliable criterion for determining drill bit breakage could be established. The proposed method offers possibilities for new measurement and analysis methods that have not been used in conventional tool wear and damage studies. The advantage of the proposed method is that the measurement device is easy to install and the measured signal is resistant to electromagnetic noise and ambient temperature because optical fiber is used as the signal transmission medium. It also eliminates the need for complex analysis of the measured signal, eliminating the need for a high-performance analyzer and reducing analysis time.

## 1. Introduction

Nowadays, in the fourth industrial revolution and the internet of things (IoT) era, the manufacturing industry is aggressively pursuing unmanned and automated processes to increase its competitiveness; automated measurement and technology for monitoring the factors affecting product quality are indispensable. Researchers have attempted to solve the significant problem of designing an automated system for a machine tool capable of measuring the state of a cutting tool and replacing it when it breaks due to wear [1,2]. Abnormal conditions that occur during cutting, such as tool wear, tool breakage, and chattering, not only reduce machining accuracy and surface quality, but also increase production time and cost due to tool replacement and rework. Therefore, it is necessary to implement prompt measures by predicting and detecting abnormal states on the cutting tool [3,4,5,6]. The methods for monitoring the state of the cutting tool can be divided into direct methods and indirect methods. In direct methods, wear can be intuitively measured by directly assessing the state of the cutting tool using a microscope or an image sensor. However, to implement this approach, it is necessary to separate the cutting tool from the machine tool, which reduces productivity due to interruption of the manufacturing process. Accordingly, many studies on indirect methods for monitoring the state of a cutting tool using various sensors have been recently conducted. In indirect methods, variables such as cutting force, vibration, acoustic emission, and current are measured. Dimla and Lister used a force sensor as a tool post dynamometer to measure all three mutually perpendicular components of cutting force. The data from the force sensor was monitored through time series and fast Fourier transform analysis, and the influence of various wear modes occurring simultaneously in the insert of the tool was shown [7]. Li used acoustic emission. Acoustic emission is one of the most effective ways to detect tool wear. Acoustic emission can be used for monitoring because the frequency range of the signal is much higher than that of mechanical vibration and ambient environmental noise [8]. Alonso and Salgado conducted condition monitoring based on tool vibration signals using singular spectrum analysis and cluster analysis [9]. Singular spectrum analysis analyzed and predicted the obtained tool vibration signal, and Rao et al. measured and predicted the vibration signal using the Doppler vibration example [10]. Salgado and Alonso used a motor current to monitor the condition of the tool [11]. Tool wear was predicted by monitoring power consumption signals such as by Al-Sulaiman [12]. Kulkarni et al. monitored using the tool working thermocouple method to determine the thermal magnetic field signal generated at the thermal junctions generated by interactions between the workpieces. It was confirmed that high temperature occurred at the edge of the tool, and the wear rate of the tool affected the temperature [13]. Abouelatta et al. made predictions by measuring the surface roughness [14]. Wang et al. studied the correlation between flank wear of drill, maximum drilling torque, and surface roughness of machined holes [15]. Kuntoğlu et al. studied that the condition of the tool was monitored by applying the sensor fusion method to acoustic emission, current, temperature, force, and vibration signal [16]. Mohanraj et al. studied condition monitoring for end milling process using wavelet features and Hoelder’s exponent with machine learning algorithms [17]. Dahe et al. studied the tool condition monitoring with vibration signal using fuzzy unordered rule induction (FURIA) and random forest algorithm [18]. Jaini et al. studied tool monitoring of end milling based on gap sensor and machine learning [19]. Zhang et al. predicted flank wear with a hybrid model using convolutional neural networks (CNN) and long short-term memory (LSTM) models for vibration signals, acoustic emission, and force signals [20].

However, indirect methods present several disadvantages, such as the need for expensive equipment, complex signal processing, and difficult installation [7,14,21,22,23,24,25]. To overcome the limitations of direct and indirect methods, a novel method for monitoring the condition of the cutting tool is proposed in this paper; a low-cost commercially-available infrared sensor is used to measure the abnormalities of the cutting tool while the machine tool is in operation and thus minimize the interference on the machining process. Moreover, this method is validated experimentally. The cutting tool used for the application and validation of the proposed method is a drill bit; drill bits are important cutting tools, as approximately 25% of machining processes employ this element [26]. In addition, drilling can generate a significant economic loss if a product defect occurs at the end of the machining process, in a subsequent step. Therefore, a tool life prediction method for a drill is proposed in this paper.

## 2. Theoretical Background

### 2.1. Drill Bit Wear and Tool Life Criteria

**Figure 1:**
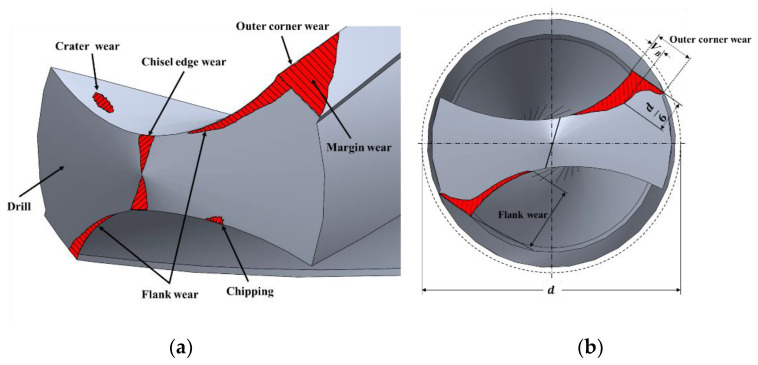
(**a**) Classification of drill bit wears; (**b**) comparison between flank wear and outer corner wear.

Drill bit wear can be of many types, such as flank wear, crater wear, outer corner wear, margin wear, chisel edge wear, and chipping, as shown in Figure 1a [27]. In many studies, flank wear has been used as a drill life criterion as wear appeared on the flank of the drill in the direction opposite to the cutting direction due to friction between the flank and the workpiece. As shown in Figure 1b, when the flank wear width (*V_B_*) measured at a point 1/6 of the diameter away from the outermost margin of the drill reaches approximately 300 µm, the drill bit should be replaced [24,26]. Flank wear can be easily measured only for a relatively simple cutting tool such as a bite; it is difficult to measure flank wear on a tool with a complex shape such as a drill bit or an end mill. Therefore, for measuring the flank wear width on a drill bit, it is necessary to remove the drill bit from the machine tool and perform a direct measurement using a microscope or an image sensor [26]. However, non-contact optical equipment is difficult to use and, for machine tools, expensive confocal lenses and complex optical systems are required. As for flank wear, corner wear has been found to be directly related to drill life by many researchers [26,27]. As corner wear is easier to measure than flank wear, in the method for predicting the breakage of a drill bit proposed herein, drill bit corner wear is measured to develop an easy to implement a system for monitoring the condition of the drill bit without the need to remove the cutting tool from the machine.

### 2.2. Working Principle

**Figure 2:**
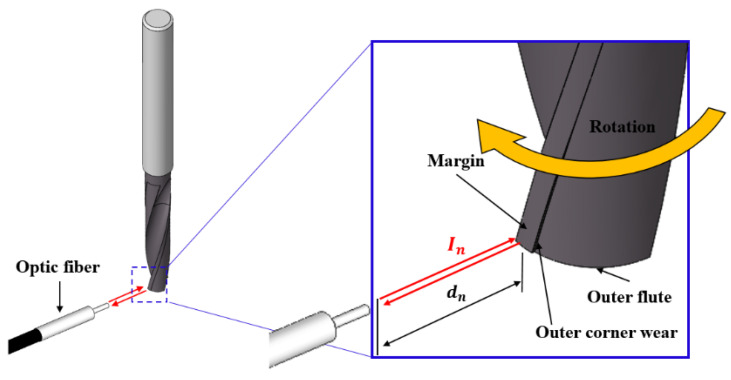
Method of measuring outer corner wear using an infrared emitter and detector.

This section describes the working principle of the system for monitoring the condition of the drill bit by measuring corner wear while the drill is operating, using a low-cost commercial infrared sensor, and optical fiber. The rotational speed of the drill bit is higher at the corner, as it increases in the radial direction. Therefore, at the corner where the rotational speed of the drill bit is highest, a significant amount of heat is generated due to the effect of the cutting speed and the friction with the wall of the machined hole during the cutting process, inducing rapid abrasion and breakage. As the number of machined holes increases, corner wear increases, and the shape and surface roughness of the corner change rapidly. The principle of an infrared sensor is based on the measurement of the intensity of light reflected from an obstacle. Figure 2 presents a schematic of the drill bit corner wear measurement principle using an infrared sensor. If the arbitrary distance between the optical fiber and the drill is $d_{n}$, as shown in Figure 2, let the intensity of the reflected infrared light be $I_{n}$. The optical fiber was used as an optical path for infrared light so as to receive only light reflected vertically at the drill bit corner. When the position of the optical fiber is fixed, as corner wear and margin wear progress, the minute distance between the optical fiber and the drill bit corner increases, and the roughness of the drill bit surface increases, causing irregular reflection. Therefore, the intensity of light reflected from the drill bit corner and incident on the optical fiber decreases due to the micro-distance difference and the diffuse reflection caused by wear, causing a change in the output voltage of the infrared light receiving sensor. When fixing the position of the fiber and rotating the drill bit at an arbitrary distance, the measured voltage corresponding to the light reflected from the drill bit corner without wear remains unchanged. On the other hand, if wear occurs at the outer corner of the drill bit, the measured voltage changes. Therefore, the drill bit state can be monitored by using the voltage signal change of the infrared sensor due to drill bit corner wear.

## 3. Experimental Procedure

### 3.1. Workpieces and Cutting Tools

The workpiece used in the experiments was made of titanium alloy Ti6Al4V and its size was 95 × 100 × 50 mm. In order to minimize the effect of the defects or roughness of the workpiece surface on the drill bit and thus consider only the wear caused by drilling during the experiments, the Ti6Al4V workpiece surfaces were flattened with an end mill. In addition, the drill bit used in the experiment (DH500050, YG-1 Co., Ltd., Incheon, Korea) was a carbide drill bit with a 5-mm-thick TiAlN coating. Since TiAlN presents desirable properties such as low thermal conductivity and high oxidation and abrasion resistance, it was regarded as a suitable material for cutting Ti6Al4V, which presents low thermal conductivity and high strength. The specifications of the drill bit and the physical and mechanical properties of the workpiece used in the experiments are shown in Table 1 and Table 2, respectively.

### 3.2. Experimental Setup

**Figure 3:**
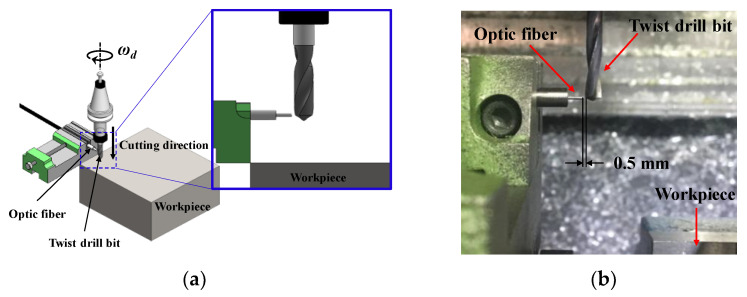
Experimental setup: (**a**) schematic, and (**b**) photograph.

**Figure 4:**
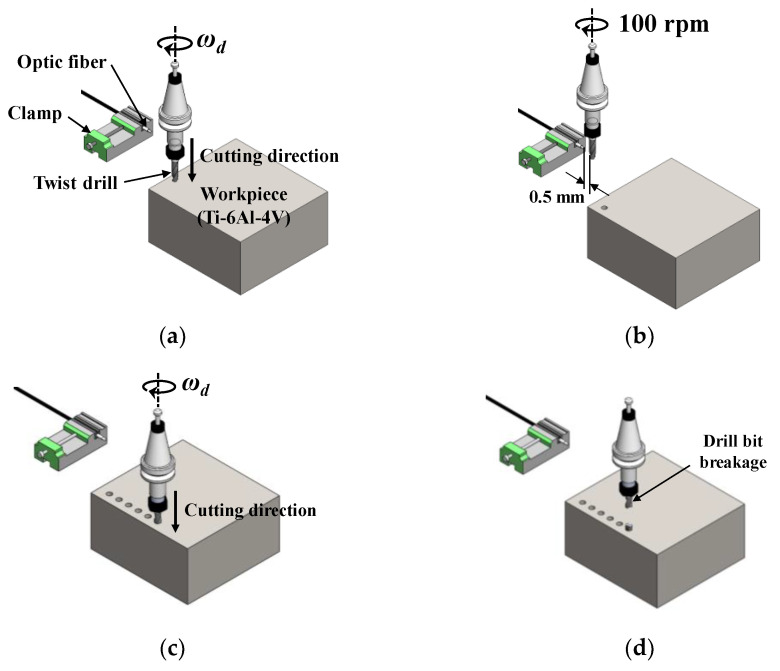
Schematic of the experimental procedure: (**a**) drilling step, (**b**) drill bit checking step, (**c**) drilling step, and (**d**) drill bit breakage.

Figure 3 presents a schematic and the device configuration for the tool breakage prediction experiment using an infrared sensor. The optical characteristics of the infrared emitter (TSAL4400, Vishay Intertechnology, Inc., Pennsylvania, United States) and infrared detector (SFH309FA, OSRAM Opto Semiconductors GmbH., Regensburg, Germany) used in the experiment are shown in Table 3. Figure 4 presents a schematic of the experimental procedure for the validation of the drill failure detecting method sensor proposed herein. As shown in Figure 4a, one optical fiber remains fixed at a specific position, and the drill produces the first hole at the spindle speed of $\omega_{d}$. After drilling the hole to the depth set on the vertical machining center and before drilling the next hole, the drill moves to an absolute position 0.5 mm away from the optical fiber and its spindle speed is 100 rpm, as shown in Figure 4b. Infrared sensors need to be used to detect changes in the texture of the wear of the outer edge of the tool, so the lower the rpm, the more accurate the waveform can be measured. Since there is a possibility that the atmosphere around the tool may be greatly distorted due to the high temperature generated by dry cutting, the measurement was performed after the tool was cooled for a sufficient time after cutting to prevent distortion of measurement data due to high temperature. After rotation at 100 rpm for 3 s, drill corner wear is measured using an infrared sensor. In this operation, the infrared photons reflected from the drill bit corner and measured by the light receiving sensor are converted into voltage signals through an electric circuit and stored in a computer via a data acquisition (DAQ) board. After corner wear measurement, the drill is moved to the designated position manually, as shown in Figure 4c, to perform the drilling process again; this process is repeated until before the drill bit breaks, as shown in Figure 4d. The experimental conditions for the validation of the drill failure prediction method sensor proposed in this paper are shown in Table 4. The above experiment was repeated three times under each experimental condition.

## 4. Experimental Results and Discussion

### 4.1. Relationship between Surface Roughness and Breakage

**Figure 5:**
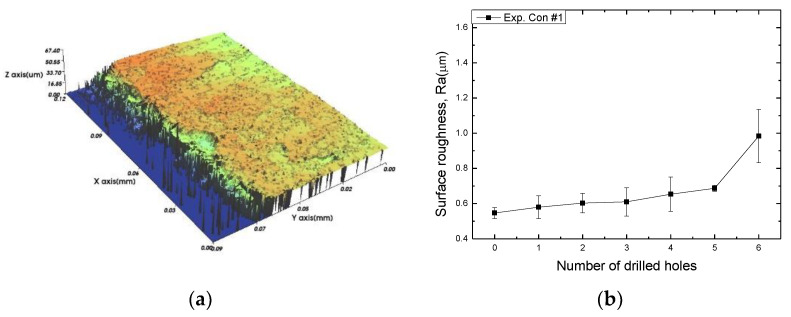
Surface roughness of the outer corner of the drill bit as shown in Figure 3: (**a**) three-dimensional surface image before drill bit breakage (after drilling the 6th hole); (**b**) surface roughness of the outer corner of the drill bit as a function of the number of drill holes.

**Figure 6:**
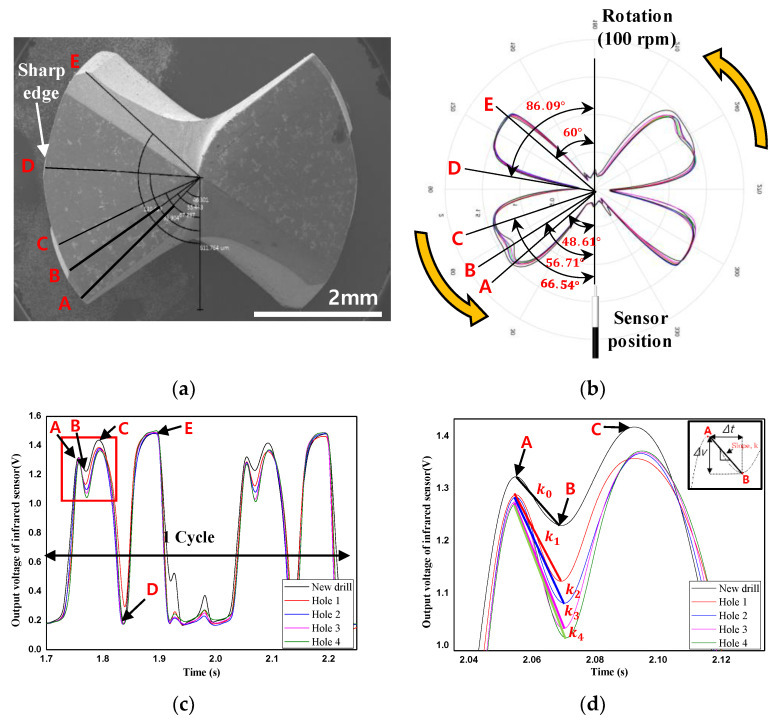
(**a**) Scanning microscope micrograph of the TiAlN-coated carbide twist drill bit before use. (**b**) Polar axis graph of reflected infrared light intensity during one drill bit rotation at 100 rpm. (**c**) Time-domain graph of reflected infrared light intensity during one drill rotation at 100 rpm. (**d**) Magnification of (**c**).

In this paper, the drilling process was carried out in dry processing conditions, i.e., without cutting oil, to accelerate drill wear and breakage. When the drilling process was carried out, as indicated in Figure 5, while drilling the seventh hole under experimental condition 1, the sixth hole under experimental condition 2, the fifth hole under experimental condition 3, and the sixth hole under experimental condition 4, the drill bit broke, respectively. Table 5 shows a scanning electron microscope (S-4800, Hitachi High-Technologies Corporation, Tokyo, Japan) image of the drill bit corners before drill bit breakage for each experimental condition; as the number of drilled holes increases for each experimental condition, the edge of the drill bit corner collapses, corner wear occurs (corner dents appear), and surface roughness increases. The surface roughness of the drill bit corner was measured using a 3D surface profiler (NVC 0505, Nanosystem Co., Ltd., Deajeon, Korea) to quantitatively evaluate the change in surface roughness as the number of drilled holes increased. Surface roughness was calculated using the mean and standard deviation from measuring the centerline average roughness, Ra, three times according to the number of drilled holes. Figure 6b shows the mean and standard deviation of the center line average roughness as a function of the number of drilled holes under experimental condition 1 (Table 4); Ra tends to increase as the number of drilled holes increases, and to increase rapidly immediately before the drill bit breaks. In addition, Figure 6a presents three-dimensional values of Ra measured at the hole drilled immediately before breakage, under experimental condition 1. By comparing the scanning electron micrographs of the drill bit corners with the experimental results by the three-dimensional surface measuring machine, it was confirmed that the shape and surface roughness of the drill bit corner immediately before drill bit breakage changed drastically and diffuse reflections occurred on the drill bit corner surface. Consequently, the diffuse reflection at the corner immediately before drill bit breakage caused a change in the voltage signal measured by the infrared light receiving sensor.

### 4.2. Reflected Infrared Light and Outer Corner Wear

In order to experimentally validate the drill failure prediction method proposed in this paper, drill bit corner wear was measured as the number of drilled holes increased following the procedure shown in Figure 4 under the conditions shown in Table 4. Based on the above conditions and procedures, the threshold for drill failure was set based on the voltage signal of the infrared light receiving sensor. Figure 6c shows the voltage signal measured by the infrared sensor during one rotation of the drill when the infrared sensor module is fixed and the drill bit rotates at 100 rpm (Figure 4b), and under experimental condition 3 (Table 4). In Figure 4b, the voltage signal waveform measured by the infrared sensor changes at five inflection points: A, B, C, D, and E. The section where the voltage waveform changes linearly with the number of drilled holes is that between points A and B; an enlarged graph of these two inflection points is shown in Figure 6d. The mean and standard deviation of the slopes (Figure 6d) between points A and B calculated by three drill bit corner wear measurements were −1.24 ± 0.16, −3.56 ± 0.08, −4.07 ± 0.13, −4.14 ± 0.13, and −5.87 ± 0.05, for one, two, three, four, and five drilled holes, respectively. The differences between the aforementioned slope means were 2.31, 0.31, 0.07, and 1.72 (i.e., 2.31 is the absolute value of the difference between the mean value measured after the first hole was drilled and the mean value measured after the second hole was drilled). The slope change was largest for the first hole, gently decreased as the number of drilled holes increased, and then rapidly increased to 1.72 between the fourth and the fifth hole (before drill bit breakage). This is because, as mentioned in Section 4.1 for the drill failure mechanism, the intensity of light reflected from the corner of the drill bit changes rapidly due to the diffuse reflection caused by the sharp change in shape and roughness of the drill bit corner immediately before drill failure. In order to determine whether the slopes between points A and B are related to drill bit corner wear, the infrared sensor signal for one rotation of the drill bit was converted to polar coordinates, as shown in Figure 6b. By this means, the position of points A and B can be expressed in degrees from the position of the fixed infrared module. The calculated position for points A and B were 48.61° and 56.71° from the infrared sensor module, respectively. These results can be verified from Figure 6a, in which a scanning electron micrograph taken in the vertical direction by cutting the TiAlN-coated drill bit used in the experiment is presented. Therefore, it was confirmed that points A and B corresponded to the drill corners in the voltage signal of the infrared sensor; the possibility of performing drill failure prediction using the sudden slope change immediately before drill bit breakage was also confirmed.

**Figure 7:**
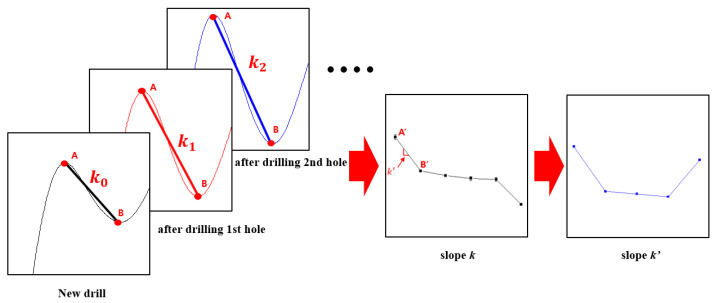
Data processing procedure for drill bit breakage prediction.

Figure 7 presents a schematic of the method for predicting drill failure through the rate of change of the slope by using the average value of three times the slopes between points A and B for different numbers of drilled holes under each experimental condition. By looking at the waveform of the slope in Figure 6d after the first hole was drilled, the slopes between points A and B suddenly change, and this change gradually proceeds as the number of drilled holes increases.

This result is due to the drill failure mechanism in which the change of the drill corner after machining the first hole increases and wear gradually increases as the number of drilled holes increases, causing diffuse reflection due to the sharp change in shape and roughness of the drill bit corner immediately before drill bit breakage. Based on this mechanism and the voltage signal of the infrared sensor detector, an arbitrary slope vector, as shown in Equation (1), was defined in order to minimize the changes produced by the machining and measurement environment and thus accurately predict the drill failure.

(1) $$\vec{S_{n}}=\left(x_{n}-x_{n-1},\;y_{n}-y_{n-1}\right)$$

**Figure 8:**
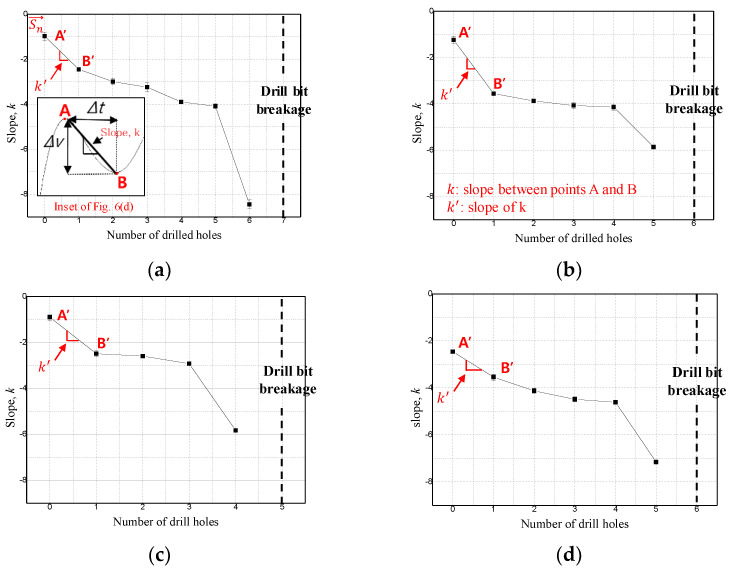
Slope between points A and B, k, from Figure 6d as a function of the number of drilled holes: (**a**) experiment condition 1, (**b**) experiment condition 2, (**c**) experiment condition 3, (**d**) experiment condition 4.

**Figure 9:**
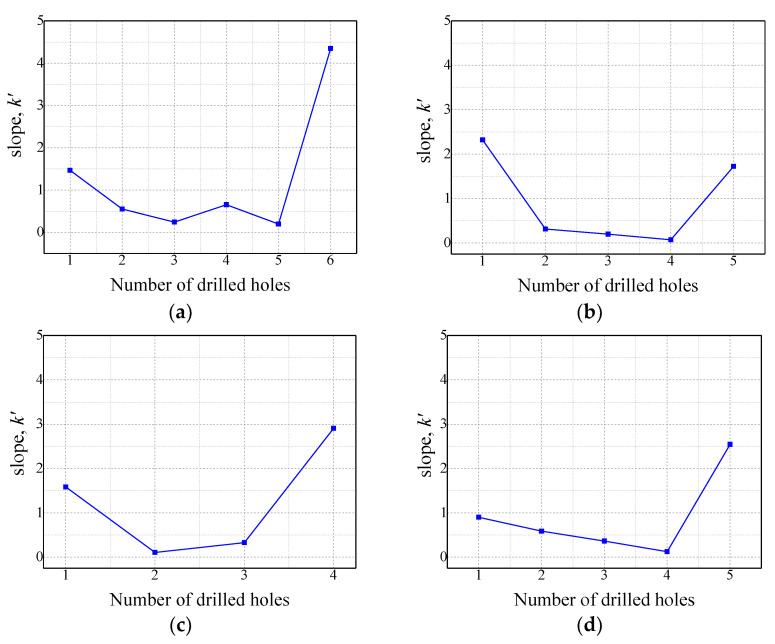
Slope between points A’ and B’, k’ (i.e., the slope k in Figure 8): (**a**) experiment condition 1, (**b**) experiment condition 2, (**c**) experiment condition 3, (**d**) experiment condition 4.

In Equation (1), *n* is the hole machining order (*n* ≥ 1), $\vec{S_{n}}$ is the inclination vector for the nth hole (Figure 8), $x_{n}$ is the nth hole, and $y_{n}$ represents the inclination of two points, A and B, for the nth hole. Figure 9 shows the ratio of the x-direction component, the y-direction component, and the x-direction and y-direction components of $\vec{S_{n}}$ under each experimental condition of Figure 9. In Figure 9, since the slope changes in the negative direction, the components in each direction are expressed as absolute values to facilitate the comparison between the x and y components. The experimental results show that the x- and y-direction components of $\vec{S_{n}}$ are reversed for the hole immediately before breakage. This is because, as shown in Figure 9a, the arbitrary angle, θ, of the tilt vector is sharply tilted toward the negative y direction just before drill bit breakage; as an abrupt change in the shape and surface roughness of the drill bit corner occurs due to a sharp increase in drill bit corner wear immediately before the drill bit breaks, the slope of the infrared light receiving sensor signal between points A and B increases rapidly. As shown in Figure 9, the ratios of the x- and y-direction components of the gradient vector before drill bit breakage calculated for experimental conditions 1, 2, 3, and 4 are 4.35, 1.72, 2.91, and 2.54, respectively. This implies that the slope vector immediately before breakage is inclined by at least 45° with the negative y-direction component. Therefore, in this paper, the nth hole, whose ratio between the x- and y-direction components of the gradient vector is greater than 1, is determined as the drill failure prediction point.

## 5. Conclusions

In this paper, a method for measuring drill bit corner wear using a low-cost commercial infrared sensor, and thus predicting the breakage point of a drill bit based on signal change, was proposed and experimentally validated. The following conclusions can be drawn from this study:

1. Drill bit corner wear increases as the number of drilled holes increases. Scanning electron micrographs and three-dimensional surface topography results showed that the shape and surface roughness of the drill bit corners change drastically immediately before the drill bit breaks.
2. Changes in drill bit corner shape and surface roughness result in diffuse reflection, causing changes in the light intensity measured by infrared sensors. The change in light intensity is represented by the change in the slope between inflection points A and B of the voltage signal measured by the infrared sensor.
3. By converting the time–voltage waveform measured during one rotation of the drill bit to angle–voltage polar coordinates, the position of the two inflection points, A and B, can be expressed as the angle from the fixed infrared sensor position. The position of points A and B were found to be 48.61° and 56.71° sensor, respectively, and were confirmed by scanning electron micrographs. Therefore, the inclination change of points A and B is caused by the change in shape and surface roughness of the drill bit corner as the number of drilled holes increases.
4. The experimental results were reviewed through Liu et al. and Wang et al.: Liu et al. studied the correlation where the drilling torque increased as the outer corner increased [26], and Wang et al. confirmed the tendency of increasing the surface roughness of drilled holes and drilling torque as flank wear increased [15]. This is because the cutting edge is not sharp enough to cut the base material due to the wear of the outer corner, so that the frictional force increases, and thus the cutting surface of the tool becomes more and more rough.
5. Arbitrary gradient vectors were defined and the ratio of the *x*- and y-direction components of these vectors was selected as a criterion for predicting the drill failure prediction point. Under each experimental condition, the ratios of the *x*- and y-direction components of the gradient vector were greater than 1, indicating that the slope vector immediately before breakage was inclined by more than 45° in the negative y-direction. Therefore, in this paper, the drill failure prediction point was selected as the moment when the ratio of the *x*- and y-direction components of the gradient vector of the nth hole is greater than 1.
6. This study proposes a novel measuring device that is easy to install, can be configured at a low cost (infrared sensor price of about US $2), and is resistant to electromagnetic noise and ambient temperature.
7. Since the proposed measurement method does not require complex analysis of the measurement signal, it does not require a high-performance analyzer and can reduce analysis time.

## 6. Patent

The authors have filed provisional Korea Patent 10-2019-0079073.

## Figures and Tables

**Table 1:** Specifications of drill bit used in the experiments. (DH500050, YG1 Co., Ltd.).

Coating Material	TiAlN
Diameter	5 mm
Point angle	140°
Helix angle	15°
Total length	72 mm
Shank diameter	6 mm
Shank length	40 mm

**Table 2:** Mechanical properties of the Ti6Al4V workpiece used in the experiments.

Tensile strength	974 MPa
Elongation	8%
Reduction of area	20%

**Table 3:** Characteristics of infrared emitter and detector used in the experiments.

Model	TSAL4400(Emitter)	SFH309FA(Detector)
Spectral range	840~1030 nm	730~1120 nm
Peak wavelength	940 nm	900 nm
Viewing angle	25°	12°

**Table 4:** Experimental conditions.

Number	Workpiece	Drill Diameter	Cutting Speed	Feed Rate	Hole Depth
1	Ti6Al4V	∅ 5	23.6 m/min	0.133 mm/rev	20 mm
2			31.4 m/min	0.125 mm/rev	20 mm
3			31.4 m/min	0.1 mm/rev	20 mm
4			39.3 m/min	0.1 mm/rev	15 mm

**Table 5:** Scanning electron micrographs of the outer corner of the drill bit for different numbers of drilled holes and experimental conditions.

Experimental Condition	Number of Drilled Holes							
	0	1	2	3	4	5	6	7
Experimental condition 1	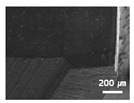	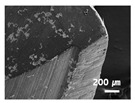	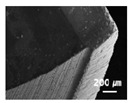	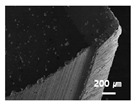	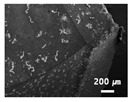	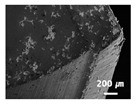	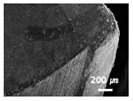	× (drill bit breakage)
Experimental condition 2	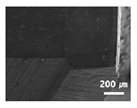	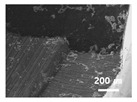	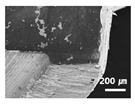	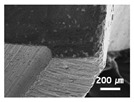	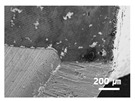	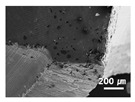	× (drill bit breakage)	
Experimental condition 3	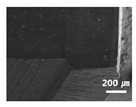	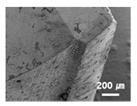	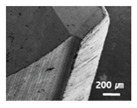	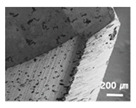	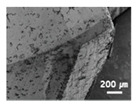	× (drill bit breakage)		
Experimental condition 4	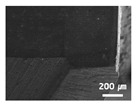	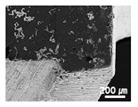	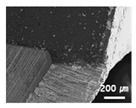	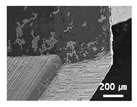	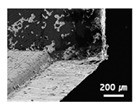	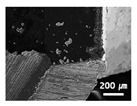	× (drill bit breakage)	

## Data Availability

Experiment data are available on request to Byeong Hee Kim.

## Nomenclature

*V_B_*	Flank wear width (μm)
$d_{n}$	Arbitrary distance between the optical fiber and the drill (mm)
$I_{n}$	Intensity of the reflected infrared light (V)
$\omega_{d}$	Rotation speed to measure outer corner wear (RPM)
$k_{i}$	Slope between A and B in Figure 8a
$k\prime_{i}$	Slope of *k* in Figure 8b
$S_{n}$	Inclination vector for the *n*th hole
